# Giant Cell Tumor of Tendon Sheath and Tendinopathy as Early Features of Early Onset Sarcoidosis

**DOI:** 10.3389/fped.2019.00480

**Published:** 2019-11-15

**Authors:** Shaoling Zheng, Pui Y. Lee, Yukai Huang, Aiwu Wang, Tianwang Li

**Affiliations:** ^1^Department of Rheumatology and Immunology, Guangdong Second Provincial General Hospital, Guangzhou, China; ^2^Division of Allergy, Immunology and Rheumatology, Boston Children's Hospital, Boston, MA, United States; ^3^Department of Pathology, Guangdong Second Provincial General Hospital, Guangzhou, China; ^4^The Second School of Clinical Medicine, Southern Medical University, Guangzhou, China

**Keywords:** giant cell tumor of tendon sheath, tendinopathy, early onset sarcoidosis, Blau syndrome, tumor

## Abstract

Giant cell tumor of tendon sheath (GCTTS) is characterized by diffuse proliferation of synovial-like cells and multinucleated giant cells along tendon sheaths. This benign tumor typically presents in the third to fourth decade of life and is exceeding rare in children. Here we describe a case of a 10-years-old girl with a history of soft tissue swelling involving the third digit of left hand, bilateral wrists and ankles. Pathology of the finger mass revealed abundant multinucleated giant cells consistent with GCTTS. Resection of the tendinous masses from the ankles also showed multinucleated giant cells along with chronic bursitis. She began to show features of polyarticular arthritis by age 7. Due to progression of arthritis, whole exome sequencing was performed and found a *de novo* heterozygous mutation in *NOD2* (p. R334Q). This variant is the most common mutation responsible for early onset sarcoidosis (EOS)/Blau syndrome, an autoinflammatory disease characterized by granulomatous inflammation of joints, skin and eyes. The early onset of symptoms and presence of multinucleated giant cells and granuloma in this case are in keeping with a diagnosis of EOS/Blau syndrome. The patient responded well to treatment with methotrexate and etanercept. This case extends the clinical spectrum of EOS/Blau syndrome, which should be considered for GCTTS and other unusual presentations of tendon inflammation in children, even in the absence of the characteristic triad of arthritis, dermatitis and uveitis.

## Introduction

Giant cell tumor of tendon sheath (GCTTS) is the second most common tumor of the hand, typically presenting in the third to fourth decade of life (1). Also known as localized nodular tenosynovitis, GCTTS is characterized by diffuse presence of multinucleated giant cells and proliferation of synovial-like cells (2). This benign lesion is predominantly found in the hand, followed by ankle-foot as the next most common site (3, 4). Tissue swelling is the typical symptom caused by GCTTS and bone erosion has been reported in advanced cases (5). Although surgical removal can be curative, recurrence of GCTTS has been reported in up to 44% of cases (6).

GCTTS is rare in children with only one large case series of 29 patients describing this entity in the pediatric population (7). Most of these patients are over 10 years of age at the time of diagnosis. Here we report a young patient that initially presented with GCTTS in the hand and tendinopathy of wrists and ankles. With progressive tendon and joint inflammation, she was ultimately found to have a classic mutation in *NOD2* responsible for early onset sarcoidosis (EOS; also known as Blau syndrome), an autoinflammatory syndrome characterized by granulomatous arthritis, uveitis and dermatitis.

## Case Description

We present a 10-years-old girl with a history of tissue swelling involving the third digit of left hand, bilateral wrists and ankles. At 2 years of age, she first developed soft tissue masses above both wrists and ankles without pain, warmth or redness. She did not receive any treatment and the soft tissue masses enlarged slowly. Two years later, she underwent surgery to remove the ankle masses and pathology was reported to be consistent with chronic bursitis. However, swelling gradually recurred near the surgical sites within 1 year.

**Figure 1:**
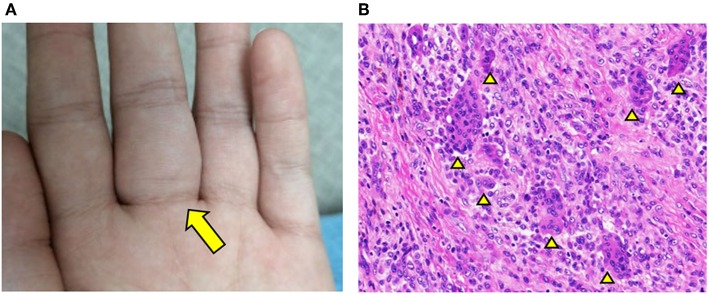
GCTTS presenting in a young child. **(A)** Presence of a soft tissue mass along the third digit of left hand. **(B)** Hematoxylin and Eosin (H&E) staining of resected finger mass consistent with a diagnosis of GCTTS. Arrowheads indicate multinucleated giant cells.

At 6 years of age, a new mass appeared on the palmar aspect of the left hand at the base of the third digit (Figure 1A). The mass was resected and histology illustrated hallmark features of GCTTS including an abundance of multinucleated giant cells (Figure 1B; see Supplementary Figures 1, 2 for full images). This diagnosis was confirmed by three independent pathologists that reviewed the histology. One year later, the patient started experiencing joint pain and gradual decline in the range of motion of wrists and ankles. Pain and swelling affected additional joints including bilateral elbows and the fifth PIP joint of left hand.

**Figure 2:**
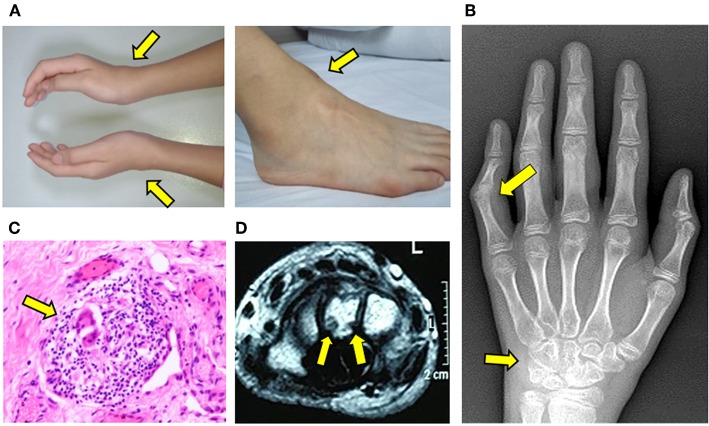
Progressive development of tendinopathy and arthritis. **(A)** Soft tissue swelling along the wrists and ankles. **(B)** X-ray of hand illustrating bone erosions of left wrist and fifth proximal interphalangeal joint. **(C)** H&E staining of resected mass from the right ankle. Arrow indicates multinucleated giant cell within a granulomatous structure. **(D)** MRI of left wrist illustrating erosions of carpal bone.

With progression of symptoms, the patient was referred to our Rheumatology Clinic for evaluation. Physical examination was notable for symmetric soft tissue masses along the anterior tibialis tendon of ankles and extensor tendons of the wrists (Figure 2A). She had tenderness and reduced range motion of those joints, suggestive of active synovitis. Laboratory investigations including complete blood count, C-reactive protein, erythrocyte sedimentation rate, ferritin, HLA-B27, T-Spot, rheumatoid factor, anti-cyclic citrullinated peptide antibodies and anti-nuclear antibodies were all unremarkable. Serum creatinine, transaminases, and lipid profile were within normal limits. X-ray showed early bone destruction of the left fifth PIP and the left wrist (Figure 2B). Musculoskeletal ultrasound revealed nodules with enhanced blood flow in the wrists and ankles. Re-evaluation of slides from her previously resected ankle masses revealed chronic bursitis with multinucleated giant cells and rare granuloma-like structures (Figure 2C).

MRI of wrists showed synovial thickening with abnormal signals around the tendon sheath, with evidence of wrist bone erosions (Figure 2D). Ophthalmology and dermatology examination did not reveal any abnormalities. Given the unusual features and progressive tenosynovitis, whole exome sequencing (WES) was performed for the patient and parents. The patient was started on etanercept (25 mg SC weekly) and methotrexate therapy (15 mg PO weekly) for possible polyarticular juvenile idiopathic arthritis while results were pending.

**Figure 3:**
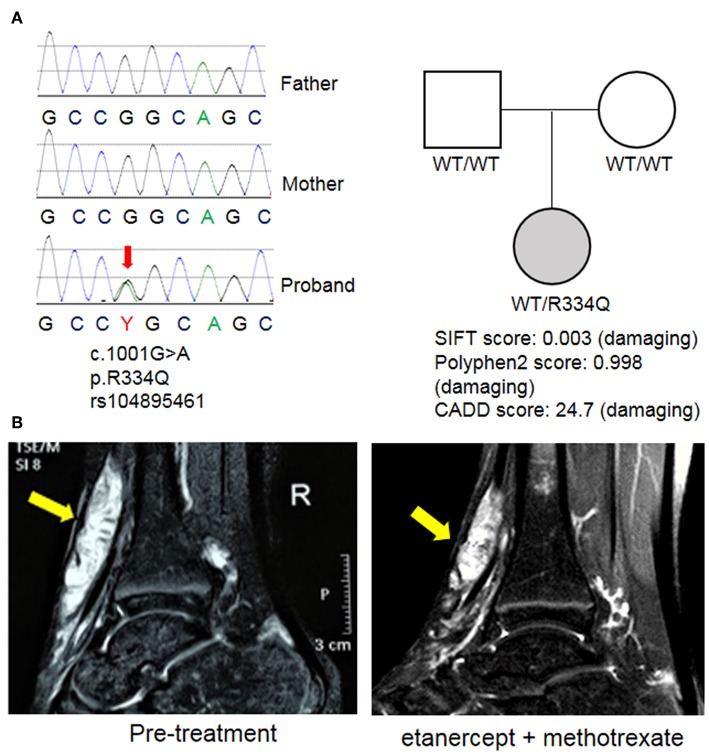
Identification of a mutation in *NOD2*. **(A)** Whole exome sequencing (WES) revealed a *de novo* mutation of NOD2 in the proband that was not found in the parents. This variant was previously described in EOS/Blau syndrome and is predicted to be pathogenic by multiple mutation analysis algorithms including SIFT, Polyphen2, and CADD. **(B)** MRI images of the right ankle before treatment and 1 year after initiation of etanercept and methotrexate. Arrows indicate an enhancing mass along the anterior tibialis tendon.

WES revealed a *de novo* heterozygous mutation in *NOD2* (nucleotide-binding oligomerization domain protein 2; exon 4; c.1001G>A; p. Arg334Gln) that was not found in either parent (Figure 3A). This mutation is a known pathogenic variant responsible for EOS/Blau syndrome (8, 9), an autoinflammatory syndrome characterized by granulomatous arthritis, uveitis and dermatitis. The *de novo* mutation and lack of family history favor the nomenclature of EOS, which typically describes sporadic cases while Blau syndrome refers to cases with familial inheritance. Given the early onset of symptoms and overlapping pathology findings, EOS likely explains the multifocal inflammation with multinucleated giant cell involvement in this patient. The patient's therapeutic response was also similar to others with EOS/Blau syndrome as treatment with etanercept and methotrexate led to steady improvement of arthritis and reduced soft tissue swelling near the wrists and ankles. After 1 year, her physical examination showed no evidence of joint swelling, joint tenderness or restricted range of motion. Marked improvement of tendinous inflammation in the anterior tibialis tendon was also demonstrated by MRI (Figure 3B), although residual enhancement was still evident despite the lack of symptoms or physical exam findings.

## Discussion

GCTTS is a slow-growing soft tissue mass that develops over months to years. The common clinical presentation is painless swelling in the hand (4, 10). The pathogenesis of GCTTS is unclear, though numerous hypotheses including neoplasia, hyperplasia, inflammatory reactions and metabolic derangements, have been proposed (1). Since most cases of GCTTS are found in adults, few studies have examined this entity in children. Gholve et al. published the only large series in children with 29 cases of GCTTS confirmed by histopathology (7). Similar to the lesions in adults, GCTTS in children are characterized by the presence of large nodules and an abundance of multinucleated giant cells in a collagenous background.

While GCTTS is typically benign and well-encapsulated, bone erosion can occur in more than $\frac{1}{4}$ of cases (1, 5). After surgical resection, 15–44% of patients experience tumor recurrence at the same site (6). Recent studies found that a high mitotic count and increased Ki-67 proliferation index in the primary tumor are risk factors for recurrence (11, 12). Cytogenetic analysis of GCTTC found that translocations involving chromosome 1p13 are present in a majority of cases. At the breakpoint of chromosome 1p13 is *CSF1*, which encodes macrophage colony stimulating factor (MCSF), a growth factor for monocytes/macrophages (13). Chromosome rearrangement in some cases leads to the fusion of *CSF1* to *COL6A3* on chromosome 2q35 (14). This rearrangement results in overexpression of a part of the MCSF protein, but whether it contributes to the pathology of GCTTS is unclear.

While the location and histopathologic characteristics of the patient's finger lesion are typical of GCTTS, she was diagnosed at an unusually young age of 6 years. Whereas most patients with GCTTS are otherwise healthy, this patient displayed progressive, multifocal tenosynovitis with evidence of granulomatous inflammation. Consistent with an autoinflammatory etiology, WES discovered a *de novo* mutation in *NOD2* associated with EOS/Blau Syndrome.

EOS/Blau syndrome is a rare autoinflammatory disorder characterized by the clinical triad of symmetric arthritis, recurrent uveitis, and granulomatous dermatitis (15). EOS is the suggested nomenclature for sporadic cases while Blau syndrome describes the familial form of the same disease (8, 16). This condition is caused by gain-of-function mutations of the *NOD2* gene that are typically inherited in an autosomal dominant manner (8, 9). The onset of disease is usually before 5 years of age, with skin rash as the most common initial manifestation. The clinical spectrum for EOS/Blau syndrome is broad and not all patients exhibit the classic triad of joint, skin and eye involvement (15). In a large cohort of 31 patients from 11 countries, arthritis was documented in all but one patient and the most common affected joints were wrists, ankles, knees and fingers (17). Cutaneous involvement and ocular inflammation each occurred in 81% of patients. Expanded manifestations beyond the classic clinical triad were seen in more than half of patients. Features including central nervous system involvement, interstitial pneumonitis, liver inflammation, vasculitis, periodic fever, and leg ulcers have been described in Blau syndrome (15, 17).

Our patient had primarily tendon involvement with initial findings of chronic bursitis and GCTTS. Her polyarticular arthritis presented later in the course and she has no history of skin rash or eye inflammation. While tenosynovitis as an extension of arthritis in EOS/Blau syndrome has been described (15), this is the first case connecting GCTTS with this intriguing autoinflammatory syndrome. Despite her unusual presentation, the patient's *de novo* NOD2 variant (R334Q) is among the most common mutations found in EOS/Blau syndrome. More than 15 mutations in NOD2 are closely associated with the disease (18). Missense mutations of amino acid residue 334 (R334W and R334Q) are observed in more than 50% of cases of Blau syndrome and no other conditions have been attributed to these variants (19). Somatic mosaicism of the R334Q mutation was also recently described (20).

How these EOS/Blau syndrome-associated mutations lead to autoactivation of NOD2 and subsequently activation of NF-κB and downstream inflammatory cytokine production remain a topic of investigation. Modeling of the disease using pluripotent stem cells showed an important role of interferon-γ in driving the inflammatory phenotype of macrophages *in vitro* (21). Recent work also revealed that the alarmins S100A12 and S100A8/9 are highly produced in EOS/Blau syndrome and the levels correlate with articular disease activity (22).

Treatment options for EOS/Blau syndrome include corticosteroids, disease modifying anti-rheumatic drugs, and/or biologics targeting tumor necrosis factor (TNF) or interleukin-1 (15, 18). Effectiveness of IL-6 blockade by tocilizumab was recently described (23). The use of TNF inhibitors seems to have the best long-term data for children with EOS/Blau syndrome (24). Thus far, our patient has responded well to methotrexate and etanercept with significant improvement in joint symptoms and function. She continues to do well with close follow up by rheumatology and ophthalmology.

## Conclusion

In summary, we present a young child with EOS that developed GCTTS and tendon inflammation early in the disease course in the absence of dermatitis or uveitis. This case suggests an intriguing link between GCTTS and EOS/Blau syndrome, two conditions that share the hallmark of multinucleated giant cells and tendon inflammation. EOS/Blau syndrome should be considered in children with GCTTS and unusual presentations of tendon inflammation even in the absence of the characteristic triad of arthritis, rash and uveitis.

## Data Availability Statement

The datasets generated for this study are available on request to the corresponding author.

## Ethics Statement

The studies involving human participants were reviewed and approved by Ethics committee of Guangdong Second Provincial General Hospital. Written informed consent to participate in this study was provided by the participants' legal guardian/next of kin. Written informed consent was obtained from the individual(s) for the publication of any potentially identifiable images or data included in this article.

## Author Contributions

SZ supervised the data collection and drafted the manuscript. PL designed the study and revised the manuscript. YH analyzed the data and drafted part of the manuscript. AW carried out the pathological analyses. TL designed the study, assigned the treatment protocol, and revised the manuscript. All authors approved the final manuscript as submitted and agree to be accountable for all aspects of the work.

### Conflict of Interest

The authors declare that the research was conducted in the absence of any commercial or financial relationships that could be construed as a potential conflict of interest.
