# Supplementary material for: Giant Cell Tumor of Tendon Sheath and Tendinopathy as Early Features of Early Onset Sarcoidosis
Source: Front Pediatr. 2019 Nov 15;7:480. doi: 10.3389/fped.2019.00480 (PMC6873213; doi:10.3389/fped.2019.00480)
Supplement: Supplementary file 1 [file Data_Sheet_1.docx]

Giant cell tumor of tendon sheath and tendinopathy as early features of early onset sarcoidosis

Shaoling Zheng^1^, Pui Y. Lee^2^, Yukai Huang^1^, Aiwu Wang^3^, Tianwang Li^1, 4*^

**Supplementary materials**

Supplementary Figure 1

Supplementary Figure 2

**Supplementary Figure 1.**


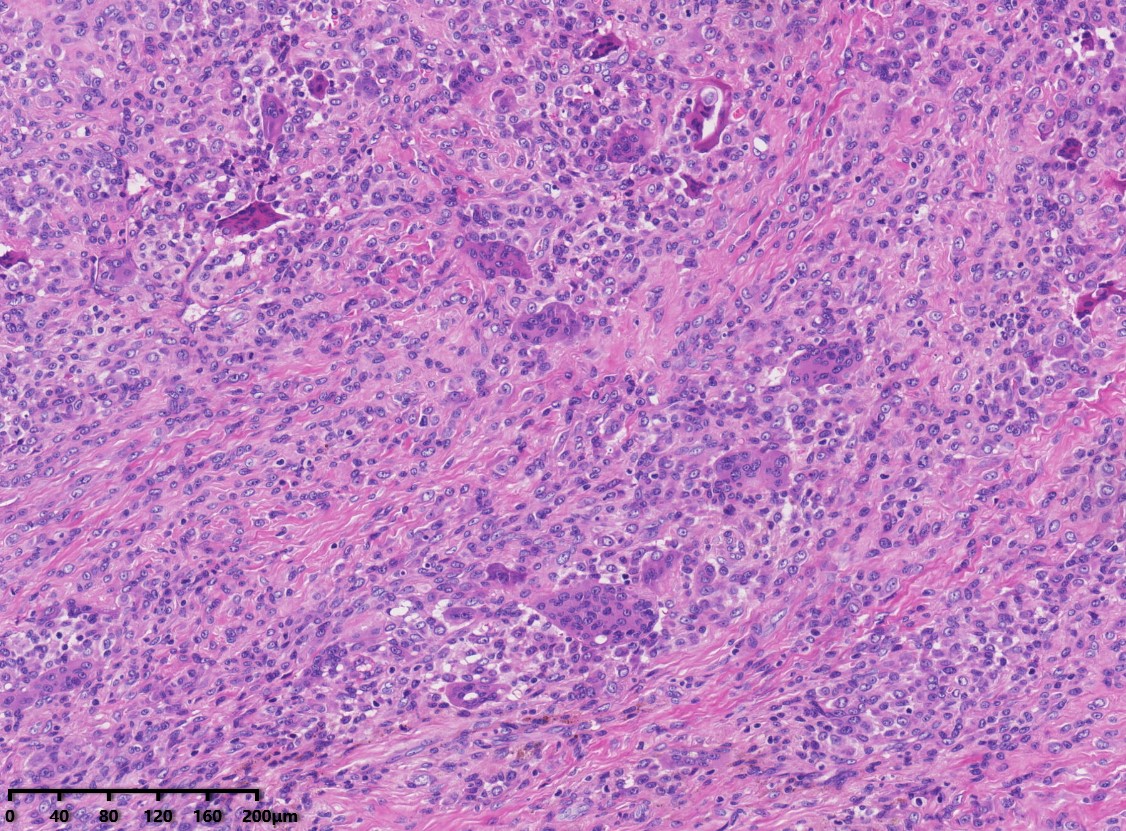


Supplemental Figure 1. H&E staining of resected finger mass with pathology suggestive of GCTTS.

**Supplementary Figure 2**


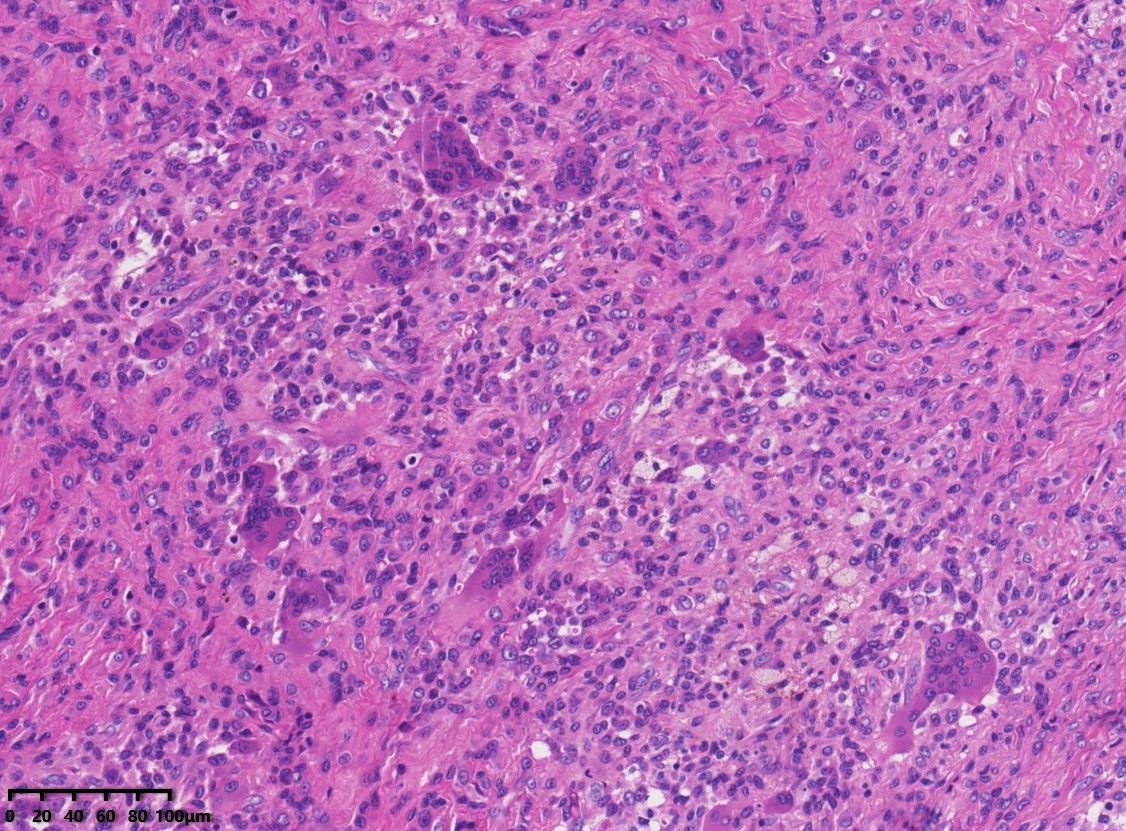


Supplemental Figure 2. H&E staining of resected finger mass with pathology suggestive of GCTTS.
